# First Principle Study of MgSnLa Compounds in Mg-3Sn-1Mn-1La Alloy Processed by Rheo-Rolling

**DOI:** 10.3390/ma15041361

**Published:** 2022-02-12

**Authors:** Jian-Hong Wang, Zhan-Yong Zhao

**Affiliations:** School of Materials Science and Engineering, North University of China, Taiyuan 030051, China; wangjianhong@nuc.edu.cn

**Keywords:** heat-resistant Mg alloy, rheo-rolling, first principle, thermodynamic properties, MgSnLa compounds

## Abstract

In order to obtain a high-performance heat-resistant Mg alloy during the rheo-rolling process, the electronic structure, elastic constants, binding energy and thermodynamic properties of the MgSnLa compounds were conducted by first-principle calculations. The results show that the MgSnLa compounds (La_5_Sn_3_, Mg_17_La_2_ and Mg_2_Sn) all show certain metallicity, and La_5_Sn_3_ has better mechanical properties (higher bulk modulus (46.47091 GPa) and shear modulus (26.40561 GPa)) than the other two phases. The binding energy reveals that La_5_Sn_3_ is the most stable phase in these composite phases (5.33 eV/atom); additionally, thermodynamic studies show that the structural stability of the MgSnLa compounds increases with the increase in temperature, and the temperature has the greatest effect on the stability of Mg_17_La_2_. These all provide an efficient guide for the widespread engineering applications of high-performance heat-resistant Mg alloy.

## 1. Introduction

Magnesium (Mg) alloys are widely used in automotive, aerospace and other fields because of their low density, excellent mechanical properties, strong anti-electromagnetic interference and excellent electromagnetic shielding ability. The main way to improve the strength and heat resistance of magnesium alloys is micro-alloying. On the one hand, microalloying could form a strengthening phase on the grain boundary, pinning the grain boundary to inhibit grain boundary slip; on the other hand, it could also play the effect of solid solution strengthening and precipitation strengthening in the matrix. Hence, the thermal stability of the strengthening phase is critical to the mechanical properties of Mg alloys.

In recent decades, introducing Sn element into Mg alloys has been proposed to form the Mg_2_Sn strengthening phase with high-temperature resistance. Additionally, its melting point (778 °C) is much higher than that of the Mg_17_Al_l2_ phase (498 °C) in traditional AZ Mg alloys, and the hardness value is up to 119 HV [1,2]. Therefore, it could be concluded that the Mg_2_Sn phase is an excellent heat-resistant Mg alloy strengthening phase [3]. Radha et al. [4] researched the microstructure, mechanical and corrosion properties of as-cast Mg-1wt%Sn-5wt% HA (hydroxyapatite) composites, and pointed out that the introduction of Sn could effectively refine the grain size and form Mg_2_Sn strengthening phase along the grain boundary, which improved the mechanical and corrosion properties of the composite alloy. Zhao et al. [5] reported the effect of Sn content on strain hardening behavior of as-extruded Mg-xSn (x = 1.3, 2.4, 3.6 and 4.7 wt%) binary alloy, and pointed out that the as-extruded Mg-Sn alloys mainly included α-Mg matrix and second phase Mg_2_Sn. Moreover, they concluded a view that Sn content weakened the strain hardening ability of as-extruded Mg-Sn alloys, but yielded an obvious elevation in its tensile strength, yield strength and elongation.

To improve the high-temperature mechanical properties of Mg-Sn alloy, some researchers added rare earth (RE) and other elements into the Mg alloy. Wei et al. [6] revealed that the feather-shaped, rod-like and massive rare earth phases formed in Mg–Sn–La alloys are the main internal factors for its better properties than as-cast Mg–5Sn alloy. Liu et al. [7] studied the microstructure and mechanical properties of permanent-mold cast Mg–5 wt% Sn–(0–2.6) wt% Di alloys (neodymium: praseodymium = 3:1), and found that the formation of Sn_x_(Nd, Pr)_y_ phase is promoted by the electronegativity difference between different elements. Moreover, they revealed that the strong segregation effect and the Sn-Di phase formed in the melt, hinder the rapid growth of crystals that would refine the grains. All these help to improve the creep resistance of the composite alloy. Pan et al. [8] investigated and compared the microstructure evolution and mechanical properties via adding Zr, Se and Ce on the as-cast Mg–3Sn–1Mn (wt.%) alloy, and concluded that adding 0.36 wt.% SC and 0.87 wt.% Ce could lead to the formation of extra phases of Mg-Sn-Sc and Mg_12_Ce, also adding 0.43 wt.% Zr or 0.87 wt.% Ce could refine the grains, these results all played a positive role in improving the tensile or creep properties of the alloy. Wang et al. [9] addressed the microstructure, tensile properties and compressive creep behaviors of the Mg-(1.65–11.52) wt.% Sn-2 wt.% Nd alloys, and dendritic α-Mg, Mg_2_Sn and Mg-Sn-Nd ternary phase could be observed. In addition, when a composition of Mg-8.23 wt.% Sn-2 wt.% Nd is determined, the highest ultimate tensile strength could be up to 140 MPa.

In the heat-resistant magnesium alloy system, Mg-Sn alloy can form Mg_2_Sn strengthening phase with high-temperature resistance and high hardness; Particularly, to further improve the high-temperature mechanical properties of Mg-Sn alloys, the researchers further found that Mn and rare earth (RE) element La play an important role in improving the mechanical properties and corrosion properties of Mg Sn alloys due to solid solution strengthening, fine grain strengthening and the formation of MgSnLa compounds [10,11]. However, considering that the structure and properties of the MgSnLa compounds are still unclear. Here, we report its structure properties through the first principle, providing an efficient guide for the widespread engineering applications of high-performance heat-resistant Mg alloy.

## 2. Computational and Experimental Procedure

**Figure 1:**
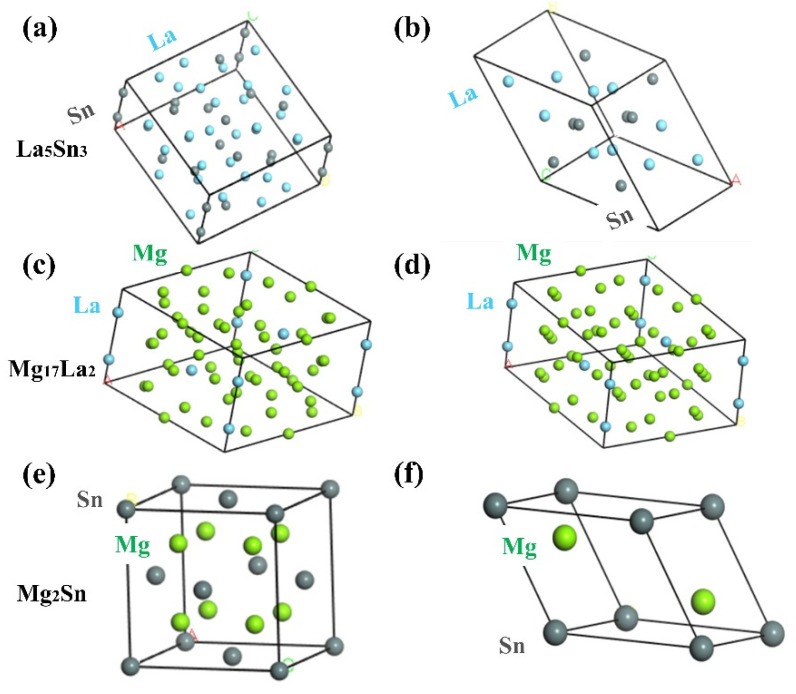
Cell models of La_5_Sn_3_, Mg_17_La_2_ and Mg_2_Sn before and after geometric optimization: (**a**,**c**,**e**) before geometric optimization and (**b**,**d,f**) after geometric optimization.

First principle calculations were performed using Cambridge Serial Total Energy Package (CASTEP) module in Materials Studio 8.0 (Accelrys, Inc. an Diego, CA, USA), which is based on the density-functional theory (DFT). It should be noted that the appropriate plane-wave energy cutoff, Monkhorst–Pack k-point grid were considered before the calculation, so as to ensure the accuracy of the calculation. Table 1 shows the crystal structure parameters of La_5_Sn_3_, Mg_17_La_2_ and Mg_2_Sn [2,12,13]. The Broyden Fletcher Goldfarb Shannon (BFGS) algorithm was used to achieve the geometric optimization of the structural model in this paper (Table 2 and Figure 1), which can accurately minimize the total energy of the system in an iterative way. Moreover, the convergence criteria containing maximum stress, maximum force and maximum displacements were set within a reasonable range.

Mg-3Sn-1Mn alloy is prepared by melting magnesium ingot (magnesium content > 99.9%), tin ingot (aluminum content > 99.9%) and manganese agent (manganese content: 80%); in particular, La element is added in the form of Mg-La master alloy. Mg-3Sn-1Mn-La alloys (15 mm × 15 mm × 10 mm) was prepared by continuous rheological rolling, and a detailed description of Mg-3Sn-1Mn-1La alloy could be found in our previous reports [11,14,15]. The specific advantages of the rheo-rolling process are as follows: (1) The vibration method effectively prevents the slurry from sticking on the surface of the inclined plate, which is efficient and convenient; (2) compared with roll casting, there is no need for complex side seal control; (3) the rolling speed is high, which is higher than the full liquid casting rolling speed.

Transmission electron microscope (TEM) characterization was performed by field-emission-gun (FEG) Tecnai G^2^ 20 microscope (FEI, Hillsboro, OR, USA) equipped with energy dispersive spectroscopy (EDS). The identification of the precipitates in the Mg-3Sn-1Mn-lLa alloys was performed in an X-ray diffraction (XRD) (X’Pert, PANalytical B.V., Almelo, Holland).

## 3. Results and Discussion

**Figure 2:**
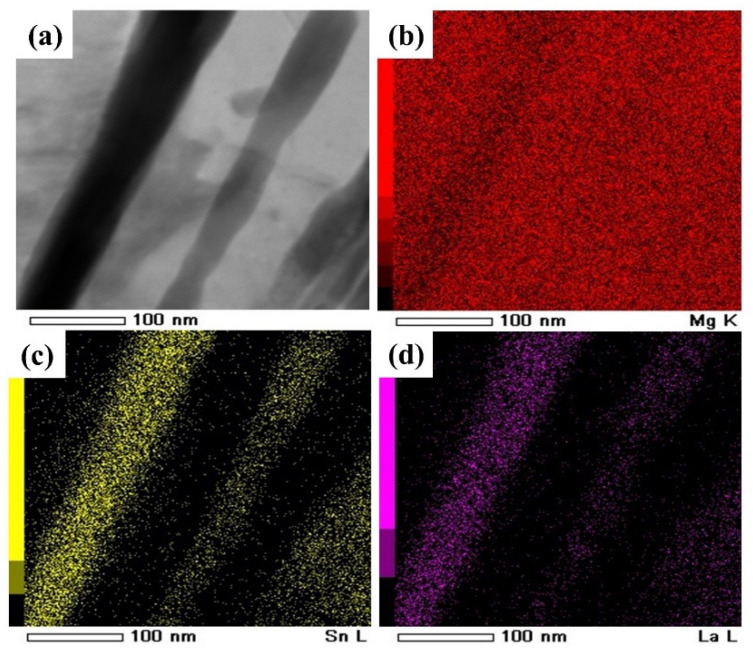
(**a**) HADDF-STEM image of Mg-3Sn-1Mn-1La alloy, (**b**–**d**) EDS map of Mg, Sn and La elements, respectively.

**Figure 3:**
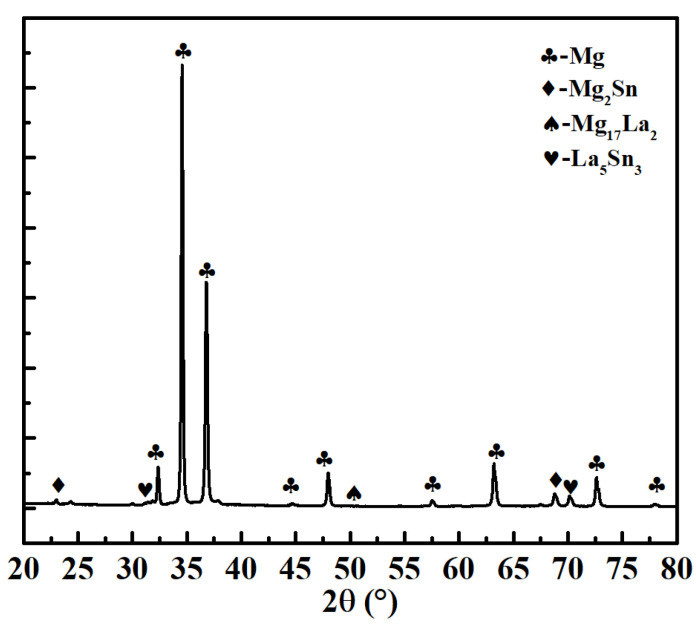
XRD pattern of the Mg-3Sn-1Mn-1La alloys showing the Mg, La_5_Sn_3_, Mg_2_Sn and Mg_17_La_2_ phases.

**Figure 4:**
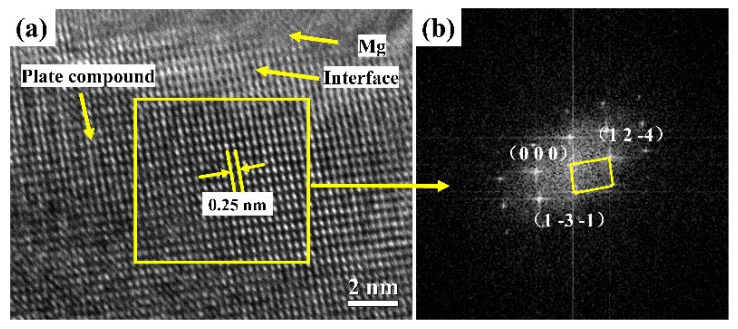
(**a**) HRTEM image of Mg-3Sn-1Mn-1La alloy, (**b**) FT map of the selective yellow box in (**a**).

Figure 2 displays a complete high angle annular dark field-scanning transmission electron microscopy (HADDF-STEM) image of the Mg-3Sn-1Mn-1La alloy where the dark area (plate-like compounds) mainly contains Mg, Sn and La elements, and XRD further proved that the plate-like compounds were composed of La_5_Sn_3_, Mg_2_Sn and Mg_17_La_2_ phases (Figure 3). Among them, the identification of the Mg_2_Sn phase was confirmed by the high-resolution transmission electron microscopy (HRTEM) and the Fourier transform (FT) pattern (Figure 4). The HRTEM of other phases (La_5_Sn_3_ and Mg_17_La_2_) have been reported in our previous studies [14]. 

**Figure 5:**
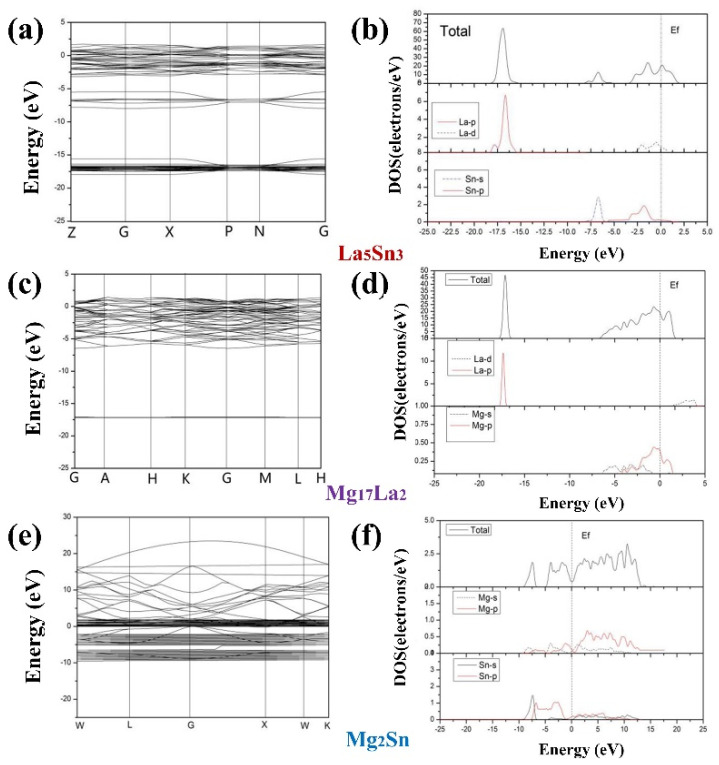
Detailed energy-band structures and density of states of La_5_Sn_3_, Mg_17_La_2_ and Mg_2_Sn: (**a**,**c**,**e**) band structures and (**b**,**d**,**f**) density of states.

In the present work, the energy band structures and density of states (DOS) are calculated to have a profound insight into the bonding of La_5_Sn_3_, Mg_17_La_2_ and Mg_2_Sn phases [16]. In Figure 5, the Fermi levels of the three phases intersect the conduction band (Figure 5a,c,e), and the partial density of states (PDOS) of La and Mg cross the Fermi level Ef (Figure 5b,d,f), indicating that the three phases have metal properties. 

Further, it could be seen that the total DOS of the La_5_Sn_3_ phase (Figure 5b) could be divided into three regions: one region ranges from −18 to 15 eV, and the DOS of this region is mainly contributed to by the 5p state of La; the other is at −7.5–6 eV, this DOS of the region is mainly contributed to by the 5s state of Sn; while the DOS at the conduction band is mainly contributed to by the 5d state of La and a small amount of 5p states of Sn. All these indicate that the strong hybridization of La 5d and Sn 5p orbitals makes a great contribution to the metal properties of La_5_Sn_3_. For Mg_17_La_2_, the total DOS is roughly divided into two regions: one region is located at 16 eV–18 eV, and the DOS in this region is contributed to by the 5d state of La; the other conduction band region is mainly contributed by the 2p and 3s orbits of Mg; moreover, it can be seen from the energy band diagram (Figure 5c) that the 5d orbital of La is a straight line, indicating that La does not participate in hybridization. However, as for the Mg_2_Sn (Figure 5e), the wide energy band and the great fluctuation of the energy band promote the strong expansibility of the atomic orbitals that make up the energy band. Meanwhile, from Figure 5f, it can be concluded that the energy band is hybrid from the s and p orbitals of Mg and the s and p orbitals of Sn.

Elastic constants play a great role in characterizing the elastic properties of materials, and occupy an important position in the mechanical properties of materials. Moreover, the calculation of crystal elastic constants is closely related to the symmetry of crystal cells, and the calculated independent elastic constants are different under different crystal systems. Hence, this paper only discussed the independent elastic constants of tetragonal, hexagonal and cubic crystal structures.

For the tetragonal crystal structure, there are six independent elastic constants: C_11_, C_12_, C_13_, C_33_, C_44_ and C_66_. The elastic stability criterion of the tetragonal crystal structure is:

C_11_ > 0, C_33_ > 0, C_44_ > 0, C_11_ − C_12_ > 0, 2(C_11_ + C_12_) + C_33_ + 4C_13_ > 0 (1)

C_66_ > 0, C_11_ + C_33_ − 2C_13_ > 0 (2)

For hexagonal crystal structure, there are five independent elastic constants: C_11_, C_12_, C_13_, C_33_ and C_44_. The elastic stability criterion of the hexagonal crystal structure is:

(3) $$C_{11}>|C_{12}|,\;(C_{11}+2C_{12})\;C_{33}\;>\;2\;C_{13}{}^{2}$$

For cubic crystal structure, there are three independent elastic constants: C_11_, C_12_ and C_44_. The elastic stability criterion of this cubic crystal structure is:

C_11_ > 0, C_44_ > 0, C_11_ − C_12_ > 0, C_11_ + 2C_12_ > 0 (4)

**Figure 6:**
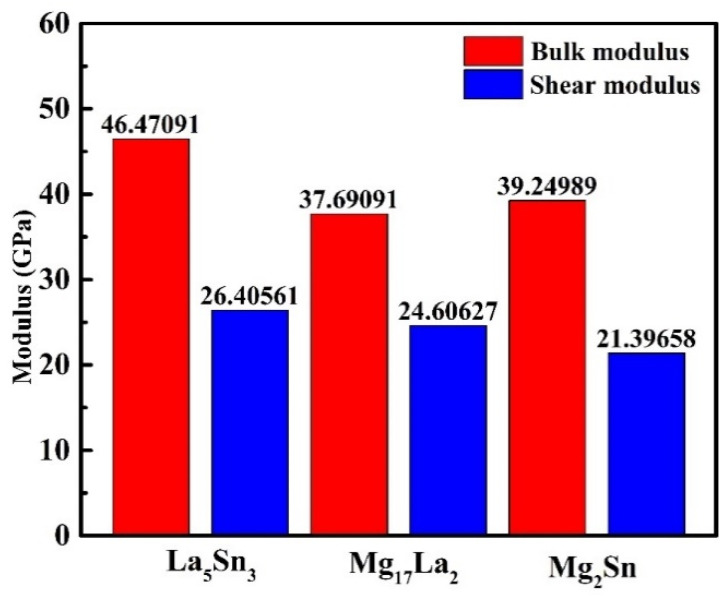
The bulk modulus and shear modulus values of La_5_Sn_3_, Mg_17_La_2_ and Mg_2_Sn.

In summary, La_5_Sn_3_ with tetragonal crystal structure, Mg_17_La_2_ with hexagonal crystal structure, and Mg_2_Sn with cubic crystal structure have been verified to meet their corresponding stability criteria (Table 3). Furthermore, it is found from Figure 6 that the La_5_Sn_3_ phase has higher bulk modulus and shear modulus values than the other two phases, indicating that the La_5_Sn_3_ phase has higher mechanical properties.

Binding energy is one of the conditions describing the stability of phase structure. It represents the energy required to split a cell structure into a single atom or the energy released when a single atom is combined into a cell. The calculation method is as follows [17]:

(5) $$E_{coh}=\frac{1}{x+y}\left(E_{tot}-xE_{atom}^{A}-yE_{atom}^{B}\right)$$

In the formula, $E_{tot}$ is the total energy of the cell, $E_{atom}^{A}$, $E_{atom}^{B}$ represents the energy of *A* and *B* free atoms, and *x* and *y* represent the number of atoms of *A* and *B* atoms in the cell structure model, respectively. The same conditions as the total energy of the intermetallic compound cell are used in calculating the free atom energy. 

On the one hand, the forming ability of the alloy can be calculated and compared by the enthalpy of alloy formation. The calculation formula is as follows:

(6) $$\Delta H=\frac{E_{tot}^{AB}-N_{A}E_{solid}^{A}-N_{B}E_{solid}^{B}}{N_{A}+N_{B}}$$

where ΔH is the enthalpy of alloy formation; $E_{tot}^{AB}$ is the total energy of intermetallic compounds; $E_{solid}^{A}$ and $E_{solid}^{B}$ represent the (average energy)/(each atom) of *A* and *B* in the solid-state; $N_{A}$ and $N_{B}$ are the number of atoms *A* and *B* in the cell.

It can be seen from Table 4 that the enthalpy of formation of La_5_Sn_3_, Mg_17_La_2_ and Mg_2_Sn is negative, indicating that these phases can be formed, and the process is an exothermic reaction. Moreover, the larger the absolute value of the enthalpy of formation, the stronger the phase formation ability [18,19]. Therefore, it can be concluded that Mg_2_Sn has the strongest formation ability, followed by La_5_Sn_3_, and Mg_17_La_2_ is the worst. For the binding energy, it refers to the energy released by combining free atoms into crystals; the larger the value of energy, the more stable the formed crystals; therefore, it can be seen that La_5_Sn_3_ is the most stable, followed by Mg_17_La_2_ and finally Mg_2_Sn.

On the other hand, the calculation of the thermodynamic performance of the system follows the standard thermodynamic statistical formula. The enthalpy (H) of the system and the Gibbs free energy (G) at each temperature are calculated by the following formula:

(7) $$H=U+\int c_{\rho }dT$$

(8) G=H−TS

where: T is the temperature; $c_{\rho }$ is the constant pressure-specific heat capacity at this temperature; U is the heat of formation at 0 K, 1.01 × 10^5^ Pa, and S is the entropy at the corresponding temperature.

**Figure 7:**
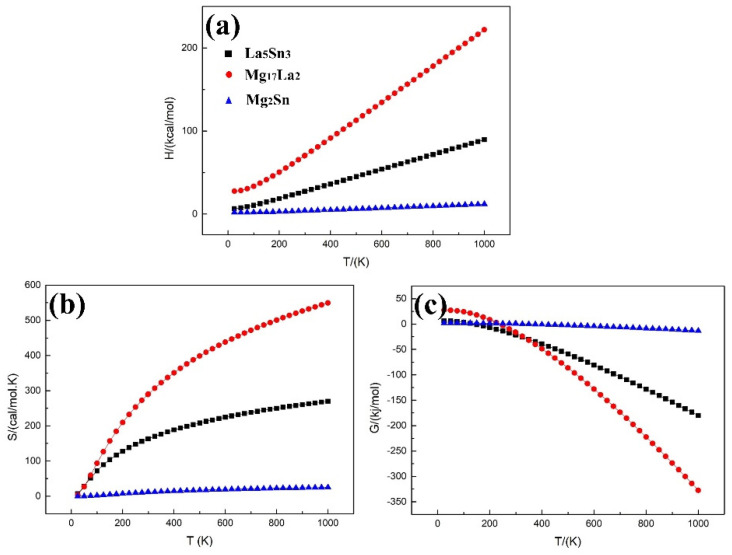
The enthalpy, entropy, and Gibbs free energy change of La_5_Sn_3_, Mg_17_La_2_, and Mg_2_Sn at different temperatures: (**a**) the enthalpy change, (**b**) the entropy change, and (**c**) Gibbs free energy change.

The quasi-harmonic Debye model is used to calculate the changes of enthalpy, entropy and Gibbs free energy of each phase with temperature [20]. When the temperature increases from 298 K (room temperature) to 1000 K, the entropy and enthalpy of the MgSnLa compounds (La_5_Sn_3_, Mg_17_La_2_ and Mg_2_Sn) increase (Figure 7a,b). On the contrary, Gibbs free energy decreases with the temperature increasing (Figure 7c), indicating that the structural stability of the MgSnLa compounds increases with the increase in temperature. Further analysis found that as the temperature increases, the free energy of Mg_17_La_2_ is most sensitive to temperature changes, and the downward trend is the largest, followed by La_5_Sn_3_, and Mg_2_Sn is the least sensitive. This may be related to the poor alloying ability of Mg_17_La_2_. This reveals that with the introduction of La, the structural stability of the Mg alloy system changes slightly with the increase in temperature; that is, the structural stability of Mg_17_La_2_ changes from less stable than Mg_2_Sn and La_5_Sn_3_ to more stable than them. Hence, it can be concluded that improving the thermodynamic stability of the alloy can be considered by increasing the temperature of the alloy. Moreover, the order of thermal stability of the three structures does not change significantly with the increase in temperature from 350 K to 675 K (Figure 7c).

## 4. Conclusions

In this study, the performance of MgSnLa compounds was compared by the first principle calculations, and the main conclusions are as follows:

(1) According to the calculation results, MgSnLa compounds (La_5_Sn_3_, Mg_17_La_2_ and Mg_2_Sn) all show certain metallicity, and La_5_Sn_3_ has better mechanical properties (higher bulk modulus and shear modulus) than the other two phases.
(2) For the binding energy, it can be concluded that La_5_Sn_3_ is the most stable, followed by Mg_17_La_2_ and finally Mg_2_Sn.
(3) The structural stability of the MgSnLa compounds increases with the increase in temperature.
(4) As the temperature increases, the free energy of Mg_17_La_2_ is most sensitive to temperature changes, and the downward trend is the largest, followed by La_5_Sn_3_, and Mg_2_Sn is the least sensitive.

## Figures and Tables

**Table 1:** Crystal structure parameters of La_5_Sn_3_, Mg_17_La_2_ and Mg_2_Sn.

Phases	La_5_Sn_3_	Mg_17_La_2_	Mg_2_Sn
Space group	14/MCM (140)	P63/MMC (194)	FM-3M (225)
Lattice constants	a = b = 12.749 Å, c = 6.343 Å	a = b = 10.35 Å, c = 10.28 Å	a = b = c = 6.81 Å
Atomic coordinates	La (0, 0.5, 0.25), Sn (0, 0, 0.25)	La (0, 0, 0.25), Mg (0.333, 0.666, 0.11)	Mg (0.25, 0.25, 0.25), Sn (0, 0, 0)

**Table 2:** Lattice constants of La_5_Sn_3_, Mg_2_Sn and Mg_17_La_2_ before and after geometric optimization.

Phases	Optimal State	a (Å)	b (Å)	c (Å)	α	β	γ
La_5_Sn_3_	before optimization	12.749	12.749	6.343	90°	90°	90°
	after optimization	9.687	9.687	9.687	96.31°	96.31°	141.3°
Mg_17_La_2_	before optimization	10.35	10.35	10.28	90°	90°	120°
	after optimization	10.43	10.43	10.16	90°	90°	120°
Mg_2_Sn	before optimization	6.81	6.81	6.81	90°	90°	90°
	after optimization	4.83	4.83	4.83	60°	60°	60°

**Table 3:** Elastic constants of Cij (GPa).

Phases	C11	C12	C13	C33	C44	C66
La_5_Sn_3_	92.47	33.56	25.13	70.99	22.09	32.36
Mg_17_La_2_	69.98	21.21	19.12	80.78	22.73	-
Mg_2_Sn	58.45	29.65	-	-	27.91	-

**Table 4:** The binding energy and enthalpy of formation of La_5_Sn_3_, Mg_17_La_2_ and Mg_2_Sn.

Phases	E/(eV/Atom)	H/(eV/Atom)
La_5_Sn_3_	5.33	−1.03
Mg_17_La_2_	2.48	−0.65
Mg_2_Sn	0.17	−6.3

## Data Availability

All the data is available within the manuscript.
